# In Situ Synthesis of Au Nanoparticles on Viscose Cellulose Sponges for Antibacterial Activities

**DOI:** 10.3390/polym11081281

**Published:** 2019-08-01

**Authors:** Mingjing Shan, Chang Liu, Lei Shi, Lei Zhang, Yuan Lin, Shuo Zhang, Zhenjun Zhu, Xiaoyin Wang, Xupin Zhuang

**Affiliations:** 1 State Key Laboratory of Separation Membranes and Membrane Processes, Tianjin Polytechnic University, Tianjin 300387, China; 2 School of Textile Science and Engineering, Tianjin Polytechnic University, Tianjin 300387, China; 3 School of Mathematical Sciences, Tianjin Polytechnic University, Tianjin 300387, China

**Keywords:** Au nanoparticles, cellulose, sponge, antibacterial activities

## Abstract

Antibacterial viscose cellulose sponges (VCSs) were fabricated by heating cellulose xanthogenate (viscose) containing HAuCl_4_·nH_2_O. Viscose was used as the reducing agent and stabilizer for the in situ synthesis of Au nanoparticles (AuNPs) onto the VCSs. The morphology, structures, thermal properties, mechanical performance, and antibacterial activities of the sponges were investigated. Results indicate that AuNPs were uniformly immobilized in the VCSs, and the resulting complexes (AuNPs@VCSs) showed enhanced thermal stability and mechanical properties. Additionally, the AuNPs@VCSs exhibited remarkable antibacterial activities, with zone of inhibition diameter of 35.7 and 37.1 mm for *Staphylococcus aureus* and *Escherichia coli*, respectively. The process is simple and applicable at the industrial level and can be applied to the fields of cleaning and sanitation.

## 1. Introduction

Sponges have low-density, high-porosity, and large specific surface areas. At present, most commercial sponges are petrochemical-based and nonbiodegradable, thereby posing challenges on environmental pollution and resource management [1,2]. Cellulose, as the most abundant natural polymer on earth, has attracted immense attention and is considered an almost inexhaustible source of raw material for environmentally friendly and biocompatible products [3]. Recently, a series of direct-solvent systems have been introduced for cellulose, including *N*-methylmorpholine-*N*-oxide (NMMO), LiCl/*N*,*N*-dimethylacetamide (LiCl/DMAc), NaOH/urea/H_2_O solution, and ionic liquids, etc. [4,5,6,7,8,9,10,11,12,13]. These systems are used in the fabrication of cellulose sponges with porous structures, which have widespread applications in oil–water separation [14], thermal insulation [15], sound insulation [16], material packaging [17], and wound dressing [18].

On the other hand, the viscose process, developed over 100 years ago, remains the widely used and prevalent cellulose dissolution process for regenerated cellulose products. In the process, cellulose is reacted with CS_2_ to cellulose xanthogenate (viscose), which can be dissolved in aqueous NaOH and convert to regenerated cellulose by using an acidic solution. Although the process was criticized for high emissions of sulfur dioxide, it has gotten effective improvements in reducing pollution and the protection of the environment in the past few decades. Moreover, in the first decade of the 21st century, the global viscose staple capacity increased from approximately 1.5 million tons (an average of 7.7% per annum) to more than 3.5 million tons, which is an all-time record high [19]. Thus, developing cellulose sponges by using viscose is practically significant. Similar to sponge-forming process in direct-solvent systems, the viscose process produces viscose cellulose sponge (VCS) when a pore-forming agent is used in the regeneration process.

Nanoparticles, especially metal nanoparticles (Ag, Cu, and Au), have widespread application in many modern technologies and have been used as antibacterial agents in several fields [20,21]. Gold nanoparticle (AuNP) is the most stable metal nanoparticle and exhibits antibacterial activity by attaching to bacterial membrane. AuNPs modify membrane potential, decrease ATP level, and inhibit the binding of tRNA to the ribosome [22]. AuNPs are highly effective against broad-spectrum microorganisms and bacterial species, viruses, and fungi. More importantly, AuNPs have considerable biocompatibility with various biomacromolecules and are nontoxic to human cells [23,24,25]. AuNPs have been incorporated into polymer matrix to acquire antibacterial ability [26]. However, polymer matrix incorporation may cause the aggregation of AuNPs owing to van der Waals interactions [27]. Applying polymers to stabilize metal nanoparticles generated by a suitable reduction method is an effective method for preventing unwanted agglomeration. In the reduction process, reducing agents, such as sodium citrate, are commonly used. Meanwhile, other oxygen-rich polymers can be used as reducing agents and stabilizers. In our previous work, microcrystalline chitosan was used to synthesize AgNPs at room temperature without any special treatment [28]. Cellulose stabilizes and reduces Au^3+^ to AuNPs in alkaline environments [29] and used in the fabrication of antibacterial cotton fabrics through the in situ synthesis of AuNPs [30,31].

Herein, a facile method for preparing composite VCSs with AuNPs (AuNPs@VCSs) by reducing HAuCl_4_·nH_2_O in situ was proposed. The AuNPs with viscose were stabilized through a hydrothermal process. The formation, structures, and properties of the AuNPs and AuNPs@VCSs were examined and discussed.

## 2. Materials and Methods

### 2.1. Materials

Cellulose (degree of polymerization of 670) was supplied by CHENGDU GRACE Co. Ltd. (Chengdu, China). Gold chloride hydrate (HAuCl_4_·nH_2_O M.W = 339.79, Au ≥ 47.5%) was provided by Tianjin Kermel Chemical Reagents (Tianjin, China). Other materials, such as carbon disulfide (CS_2_), sodium hydroxide (NaOH), and Glauber salt, were purchased from Shanghai Aladdin Co. (Shanghai, China). *Escherichia coli* and *Staphylococcus aureus* were provided by the School of Biology Science, NanKai University, Tianjin, China.

### 2.2. In Situ Synthesis of AuNPs onto VCS

Viscose was prepared using a common process. Aqueous NaOH was added to cellulose pulp with stirring, then CS_2_ was added into the mixture. Cellulose was converted to cellulose xanthogenate with CS_2_. HAuCl_4_·nH_2_O, which is equivalent to 1.6–7.9 wt.% of cellulose, was added to viscose at room temperature. The mixture was subjected to homogeneous mixing. Then, Glauber salt crystals were added. The resulting mixture was placed in a water bath at 85 ± 5 °C for 4 h and then washed. The composite sponges were coded as AuNPs@VCS-1.67, AuNPs@VCS-4.85, and AuNPs@VCS-7.83 according to the weight (1.67%, 4.85%, and 7.83%, respectively) of HAuCl_4_·nH_2_O.

### 2.3. Characterization

#### 2.3.1. Structures and Morphologies of VCS and AuNPs@VCS

The morphologies of VCS and AuNPs@VCS were characterized with a scanning electron microscope (Hitachi S-4800). XRD was performed by using an XRD spectrometer (D8 Discover with GADDS, 40 kV, 40 mA) from 10 to 80 with CuKα radiation (λ = 1.541 Å). All VCS and AuNPs@VCS were confirmed by Fourier transform infrared spectroscopy (FTIR; Nicolet 6700, Waltham, MA, USA) and X-ray photoelectron spectroscopy (XPS; Thermo-VG Scientific Ltd., West Sussex, UK).

#### 2.3.2. Performance of the VCSs and AuNPs@VCSs

A universal testing machine (Instron3369, Canton, MA, USA) was used in the extension test of the sponges at room temperature and speed of 2 mm min^−1^. In this experiment, lump-shaped samples with the volume of 10 mm × 30 mm × 3 ± 1 mm were placed in a dryer with distilled water at the bottom. Then, the samples were measured. All the samples were provided with a uniform load, and tensile strength was calculated at the linear range of the stress–strain curve. The thermal properties of the VCSs and AuNPs@VCSs were evaluated through high-resolution thermogravimetric analysis (STA409PC) from 30 °C to 500 °C at 10 °C min^−1^. Water absorption was obtained through the gravimetric method, the weights of the VCSs and AuNPs@VCSs were measured for the calculation of water absorption. Before the testing, all the sponges were uniformly cut into 15 mm × 15 mm × 3 ± 1 mm strips. The weight of each dried sample was recorded as W1 (g). All the samples were placed in distilled water at room temperature. After 12 h, the samples were removed at same time points, and excess moisture on the surface was removed with filter paper. The swollen sample weight was weighed and recorded as W2 (g). Water absorption was calculated as follows:

Water retention (%) = ((W2 − W1)/W1) × 100(1)

The water retention faculty of the sponges was determined. VCS and AuNPs@VCS sponge samples were immersed in distilled water and then transferred into centrifuge tubes containing filter papers at the bottom. The parameter of the centrifugal machine was 3 min with a rate of 500 rpm, the weight of the wet sample was weighed and recorded as W3 (g). The property of water retention was calculated by using the following formula:

Water retention (%) = ((W3 − W1)/W1) × 100(2)

The antimicrobial properties of the VCSs and AuNPs@VCSs were detected against Gram-negative bacteria *E. coli* and Gram-positive bacteria *S. aureus* through the disc diffusion method. The experimental process of disc diffusion was introduced as follows: To prepare bacterial cultures, we added bacteria to 100 mL of autoclaved nutrient broth and agitated the suspension on shaking water bath at 37 °C overnight at 160 rpm. Exactly 200 μL of the suspension was added to the as-prepared culture medium, which was dumped into a Petri dish after autoclave steam sterilization. The discs of the sponges (D = 14 mm) were placed into the culture medium for 24 h at 37 °C in an incubator. The inhibition zone diameters of the sponge samples against *S. aureus* and *E. coli* were measured [32,33].

Three specimens of same AuNPs@VCS were separately placed in bottles, which had three kinds of solutions (H_2_O, NaOH, or CH_3_COOH). Then, the bottles were fixed in shaker air bath (Julabo SW 20, Germany) and adjusted at constant shaking of 100 rpm and temperature of 30 ± 5 °C. After 48 h, all the parts of the AuNPs@VCSs extracted, and the solutions were measured by UV-Vis. Shimadzu UV-Vis spectrophotometer (UH4150) was used to qualitatively confirm the presence of AuNPs in the reaction medium. Reaction solution had an absorption range of 510–560 nm (maximum at approximately 540 nm) due to the surface Plasmon resonance band of the AuNPs [34].

## 3. Results and Discussion

### 3.1. In-Situ Synthesis of AuNPs onto VCS

**Figure 1:**
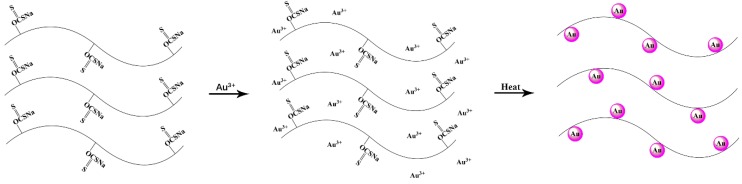
Schematic architecture of Au nanoparticles (AuNPs)@viscose cellulose sponge (VCS).

Cellulose xanthogenate is unstable and can be regenerated to cellulose in acidic environment. This information is the theoretical basis of viscose fibers [35]. Cellulose xanthogenate can also be converted to cellulose through the hydrothermal process; hence, obtaining antibacterial AuNPs@VCSs by heating viscose containing HAuCl_4_·nH_2_O is facile. Viscose is generally used as reducing agent and stabilizer for the in situ synthesis of AuNPs onto VCS, as shown in Figure 1.

**Figure 2:**
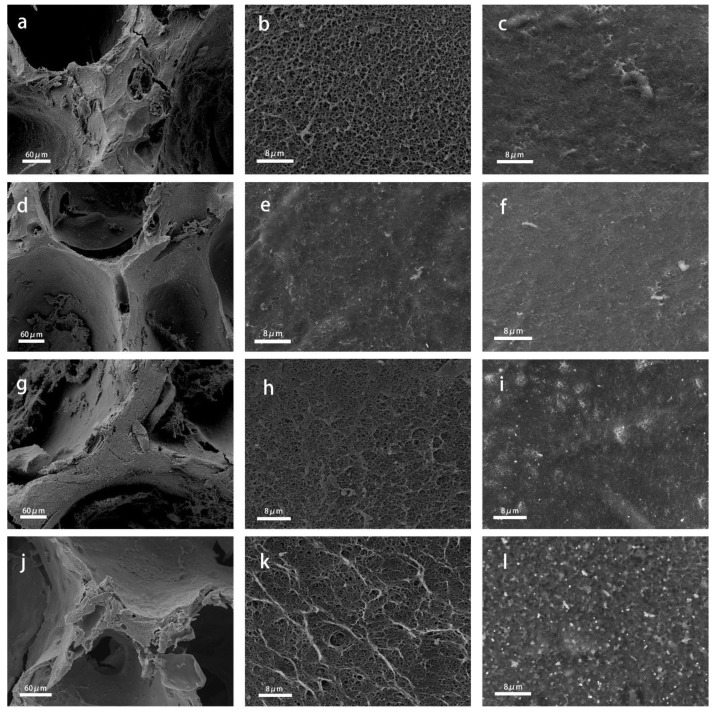
SEM images of cross profile and surface of VCS and AuNPs@VCS.

The cross profiles and surface morphology of the VCS and AuNPs@VCS samples were examined under different magnifications (Figure 2). All the cross profiles of the VCSs and AuNPs@VCSs had similar porous structures (Figure 2a,d,g,j). VCSs showed rough and clean surfaces (Figure 2c), whereas the AuNPs had uniform distributions on the surfaces of the AuNPs@VCSs after the hydrothermal process (Figure 2i,l); size increased with HAuCl_4_·nH_2_O content (Figure 2m,n). The AuNPs on the cross profile (Figure 2e,h,k) were significantly lesser than those on the surface (Figure 2f,i,l) probably because Au^3+^ had more contact area with the molecular chain of cellulose and AuNPs preferentially form on the surface.

**Figure 3:**
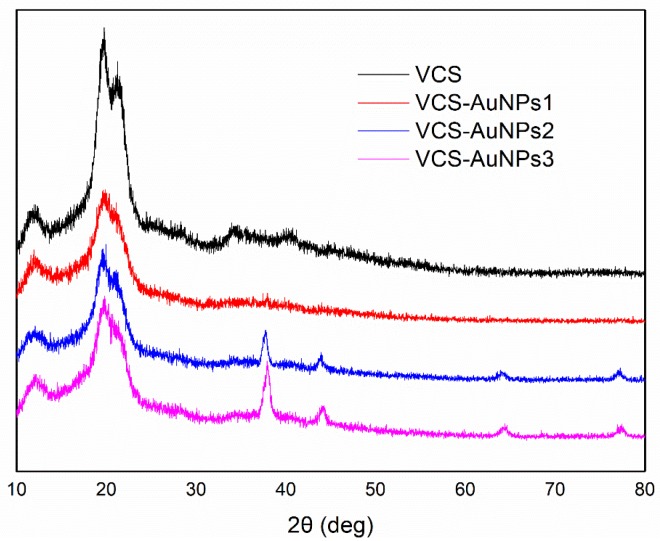
XRD analysis of VCS and AuNPs@VCS.

The effect of AuNPs@VCS crystallinity, which consequently affected VCS properties and functionality, were examined through XRD. The XRD results are shown in Figure 3. The VCS showed two intense peaks, which represents regenerated cellulose at diffraction angle (2θ = 14.6° and 21.8°), corresponding to (101) and (002) reflection of cellulose II [36]. After the AuNPs were synthesized onto VCS, four additional peaks were recorded at 2θ = 37.7°, 43.8°, 64.1°, and 77.2°, which were indexed to the (111), (200), (220), and (311) crystal planes of AuNPs [37,38]. The XRD results provided strong evidence of the presence of AuNPs.

**Figure 4:**
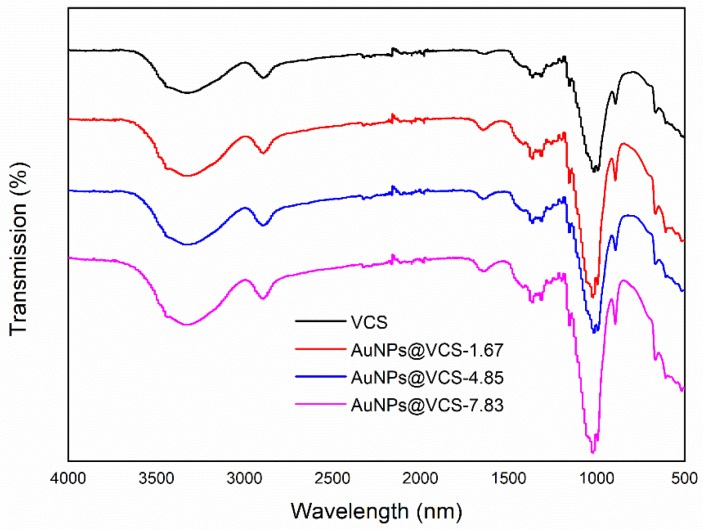
FTIR spectra for VCS and AuNPs@VCS.

**Figure 5:**
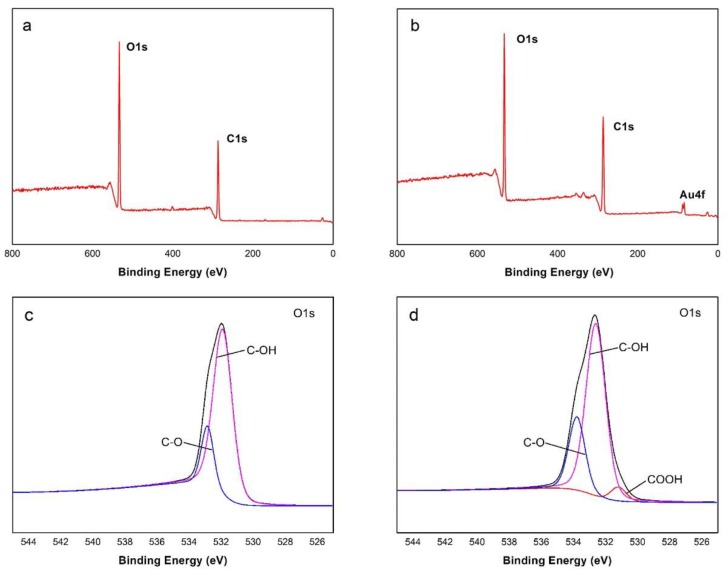
(**a**,**b**) XPS survey spectra of VCS and AuNPs@VCS. (**c**,**d**) High-resolution spectra of O1s of VCS and AuNPs@VCS.

The transmission spectra of ATR-FTIR in the VCSs and AuNPs@VCSs are presented in Figure 4. After the thermal treatment, viscose (cellulose xanthogenate) transformed to cellulose, and the three crucial absorbance peaks appeared at wavenumber of 3316 cm^−1^ (broad), 2896 cm^−1^, and 1019 cm^−1^, which were attributed to the hydroxyl (O–H), methyl (C–H), and methoxy (–C–O–) groups, respectively [39,40,41]. On the AuPNs@VCSs, new absorbance peaks were found at wavenumber of 1650 cm^−1^, which represents the vibration of carbonyl (C=O) in carboxylic groups [42]. The surface chemical compositions of VCSs and AuNPs@VCSs were further examined by XPS, and the results are shown in Figure 5. The XPS survey spectra of the VCSs and AuNPs@VCSs were observed (Figure 5a,b), and the O1s peaks were fitted by using several components (Figure 5c,d). The O1s spectra of VCS showed two peaks at 532.4 and 533.8 eV, which were related to C–OH and C–O/water. However, the O1s region of AuNPs@VCS showed one more peak than that of the VCS because of COOH at 531.6 eV [43,44]. Results of FTIR and XPS on the VCSs and AuNPs@VCSs confirmed that hydroxyl groups of cellulose were oxidized into carboxyl groups by Au^3+^.

### 3.2. Properties of VCSs and AuNPs@VCSs

**Figure 6:**
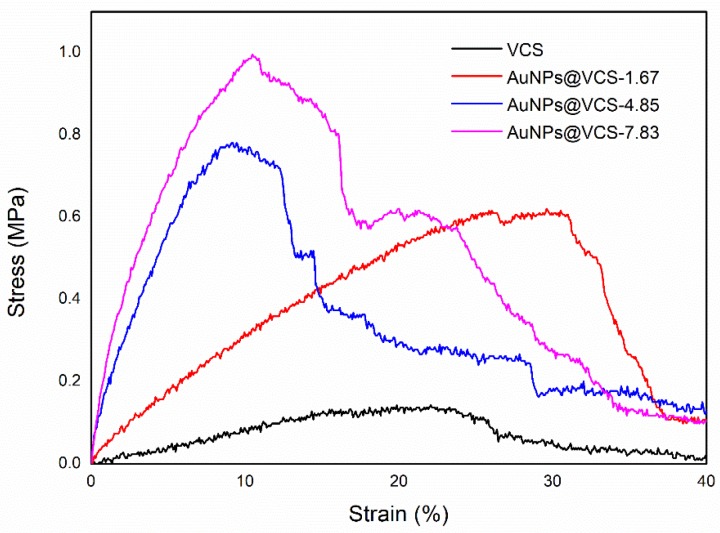
Strain–stress curves for extension test of VCS and AuNPs@VCS at room temperature.

The mechanical properties of VCSs and AuNPs@VCSs at room temperature are shown in Figure 6. The tensile strength values of the VCSs and AuNPs@VCSs were 0.15, 0.63, 0.79, and 0.98 MPa and the tensile strain values were 25.45%, 30.55%, 12.22%, and 10.39%, respectively. The findings showed that tensile strength improved with the increase in AuNPs. Strain increased first, then decreased. The results of mechanical properties show the enhancement effect of metal nanoparticles on VCSs, which is similar to the previous studies [32,45].

**Figure 7:**
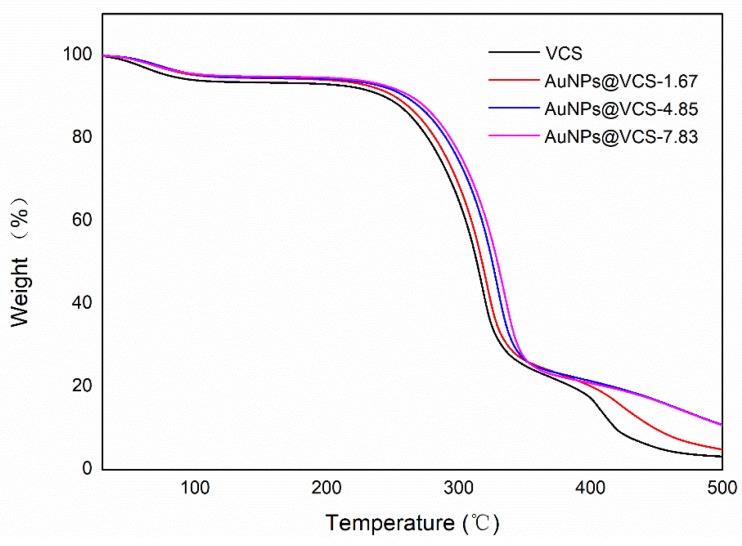
TG curves of VCS and AuNPs@VCS.

The thermostability of the VCSs and AuNPs@VCSs was examined by TG curves under nitrogen atmosphere (Figure 7). The thermal decomposition of the VCSs was conducted in three main steps. The first weight loss, approximately 6.5 wt.%, was observed from 30 °C to 100 °C and attributed to the loss of the physically bound water. The second weight loss, around 70.9 wt.%, was from 207 °C to 330 °C, showed the degradation of the side chain groups of the cellulose fibers and the breaking of the main cellulose fibers into small chains. The third weight loss, approximately 19.6 wt.%, observed from 330 °C to 500 °C, was due to the complete thermal oxidation of the cellulose chains. As shown above, the thermogravimetric curve of the AuNPs@VCS was similar to that of the VCS. However, the second decomposition step was raised to 334 °C, 339 °C, and 344 °C, which indicated the presence of thermally stable AuNPs and slightly improved thermostability of the AuNPs@VCSs. Meanwhile many previous studies have also shown similar results. The improvement of thermostability was attributed to the strong binding interaction between AuNPs and reducing polysaccharide [46,47].

**Figure 8:**
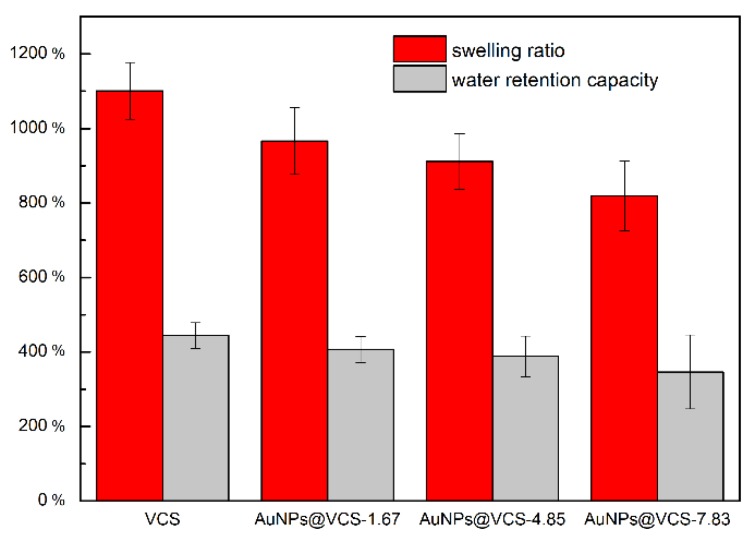
Swelling ratio and water retention capacity of VCS and AuNPs@VCS.

The efficiency of water absorption and water retention capacity are essential to antibacterial sponge. The water absorption and water retention capacities of different sponges are shown in Figure 8. The water absorption capacities of the VCSs and AuNPs@VCSs were 1101%, 996%, 911%, and 819% at increased AuNPs. Water retention capacities were 444%, 406%, 388%, and 346% (Figure 8). The water absorption and water retention capacities of VCS and AuNPs@VCS decreased gradually. This decrease indicates that water-absorbing groups, such as the hydroxy groups of cellulose, decreased because of the oxidization of Au^3+^.

**Figure 9:**
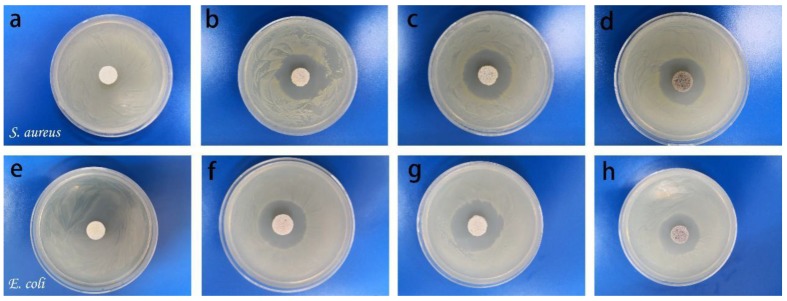
Inhibition zones of the VCS and different AuNPs@VCS against *S. aureus* (**a**–**d**) and *E. coli* (**e**–**h**) after incubation for 24 h. The diameter of sponge samples is 14 mm.

The antibacterial activities of VCS and AuNPs@VCS were measured against *E. coli* and *S. aureus* (Figure 9). VCS displayed no antibacterial activity against both bacteria, whereas the inhibition zones of AuNPs@VCS samples were observed after 24 h incubation. The AuNPs@VCSs exhibited notable antimicrobial efficacy against both bacteria. The average inhibition zone diameters of AuNPs@VCS against *S. aureus* widened from 31.4 mm to 37.1 mm and *E. coli* widened from 29.8 mm to 35.7 mm (Table 1). The distinctions of the VCS and AuNPs@VCS against Gram-negative bacteria *E. coli* and Gram-positive bacteria *S. aureus* could be attributable to the different structures of their cell wall. Compared with other antibacterial agents, AuNPs, due to their inherent physio-chemical properties, have lower biological toxicity [48,49].

**Figure 10:**
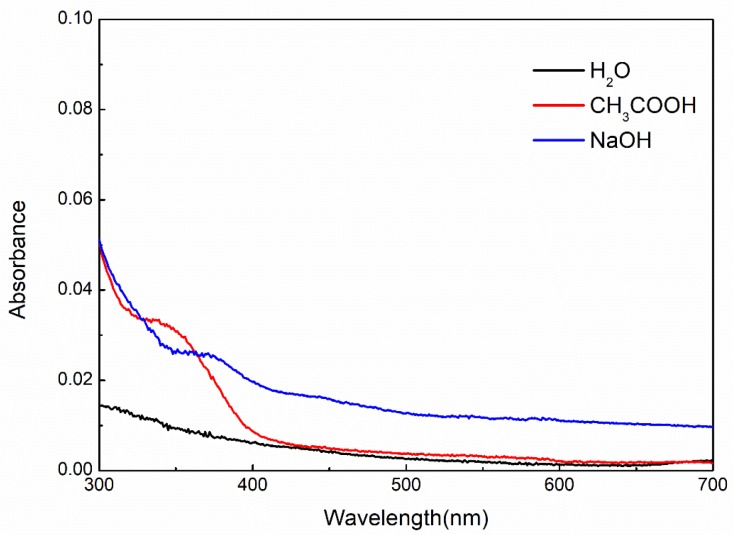
UV-Vis spectra of AuNPs@VCS-4.85 immersed in H_2_O, NaOH, and CH_3_COOH.

AuNPs@VCS were treated at different media (H_2_O, NaOH, and CH_3_COOH) and examined with ultraviolet spectrum (Figure 10) for the evaluation of the stability of the AuNPs onto VCS. The sharp peaks centered at 510–560 nm attributed to AuNPs were not observed [34]. This result indicates that AuNPs can firmly combine with VCS and have good durability on VCS.

## 4. Conclusions

Antibacterial cellulose sponges were completely formed with viscose for the in situ reduction of HAuCl_4_·nH_2_O and stabilization of AuNPs through the hydrothermal method. XRD, FTIR, XPS, and SEM confirmed the formation of the AuNPs on VCS. AuNPs@VCS exhibited remarkable antibacterial properties, and the mechanical performance, thermal stability, and stability of the AuNPs were improved. The composite sponge can provide a new antibacterial material, which can be easily fabricated at the industrial level. The results can provide references for cleaning and sanitation fields owing to the advantage of cellulose and AuNPs.

## Figures and Tables

**Table 1:** Inhibition zone diameters (mm) of sponge samples against *S. aureus* and *E. coli* after incubation for 24 h.

Bacterial Strain	Diameter of Inhibition Zone (mm)			
	VCS	AuNPs@VCS-1.67	AuNPs@VCS-4.85	AuNPs@VCS-7.83
*S. aureus*	0	33.4	35.7	29.8
*E. coli*	0	32.2	37.1	31.4
